# Vitamin D deficiency and VDR *TaqI* polymorphism on diabetic nephropathy risk among type 2 diabetes patients

**DOI:** 10.3389/fendo.2025.1567716

**Published:** 2025-06-12

**Authors:** Addisu Melake, Misganaw Asmamaw Mengstie

**Affiliations:** Department of Medical Biochemistry, College of Health Science, Debre Tabor University, Debre Tabor, Ethiopia

**Keywords:** diabetic nephropathy, *TaqI*, type 2 diabetes mellitus, vitamin D deficiency, Ethiopia

## Abstract

**Background:**

Many studies have shown that vitamin D deficiency and vitamin D receptor TaqI gene polymorphisms are associated with susceptibility to diabetic nephropathy in various populations. The objective of this study was to determine the impact of vitamin D deficiency and vitamin D receptor *TaqI* gene polymorphism on the risk of diabetic nephropathy complications in T2DM at the Debre Tabor Comprehensive Specialized Hospital, Northwest Ethiopia

**Methods:**

A total of 210 participants, including 70 diabetic patients with nephropathy, 70 diabetic patients without nephropathy, and 70 healthy controls, participated in an age—and sex-matched hospital-based case-control study. Demographic and clinical data were assessed to determine the related risk factors. DNA was extracted from blood samples and subjected to polymerase chain reaction and agarose gel electrophoresis analysis to determine the *TaqI* genotypes.

**Results:**

Vitamin D deficiency was detected in our investigation, and it was much more prevalent in diabetic nephropathy patients than type 2 diabetic patients and controls (OR = 5.05, 95% CL = 2.03–12.53; *P <* 0.001). Moreover, both the *TaqI* tt genotype (OR: 2.48; 95% CL: 1.15-5.37; P=0.020) and t allele (OR: 1.70; 95% CL: 1.13-2.57; P=0.010) were substantially more prevalent in diabetic nephropathy patients than in type 2 diabetic patients and controls, indicating that it may be a major risk factor for the development of diabetic nephropathy.

**Conclusions:**

The findings point to a potential link between vitamin D deficiency and diabetic nephropathy complications. Moreover, *TaqI* gene polymorphisms have been linked to an increased risk of developing the disease in the Ethiopian population under study.

## Introduction

Type 2 diabetes mellitus (T2DM) is a complex chronic metabolic disease that has grown to be a significant healthcare issue that killing 1.6 million people in 2015 and is estimated to rank as the seventh leading cause of death by 2030 (1). Diabetic nephropathy (DN), often referred to as diabetic kidney disease (DKD), is a major cause of morbidity and mortality in people with diabetes and a serious microvascular complication of the condition, and one of the primary causes of end-stage renal disease globally (2). It affects over 40% of T2DM patients with persistent microalbuminuria, with levels ranging from 30 to 299 mg/24 h, and is characterized by renal failure, decreased glomerular filtration rate (GFR), and increased proteinuria (3, 4). It is a multifactorial disease in which abnormal renal hemodynamic responses, hyperglycemia, hypertension, diabetes duration, hyperlipidemia, and ethnicity are some of the contributing factors that consistently increase the chance of developing diabetic nephropathy. The development and progression of DN have recently been revealed to be significantly influenced by genetic predispositions (5).

Vitamin D, a secosteroid hormone, is obtained and synthesized through diet and exposure to UV radiation (6). In addition to its well-known role in maintaining calcium and phosphorus homeostasis, it exhibits powerful non-classical features such as anti-inflammatory, antioxidant, antiangiogenic, and antiproliferative effects. Vitamin D’s non-classical functions are gaining attention due to the close relationship between vitamin D deficiency and cancer, autoimmune diseases, type 2 diabetes mellitus (T2DM), metabolic syndrome, and diabetic nephropathy, as well as the prevalence of vitamin D deficiency (7). Vitamin D has been shown in studies to be a powerful inhibitor of renal neovascularization and to reduce vascular endothelial growth factor (VEGF) production, indicating its role in the pathogenesis of diabetic nephropathy. However, there is inadequate information to determine if blood vitamin D insufficiency is associated with diabetic nephropathy, and this association has been rarely investigated (8).

The active form of vitamin D works on a specific vitamin D receptor (VDR), which is found in many human tissues and organs, including the kidney (9). The VDR is a nuclear hormone receptor superfamily member that regulates target gene transcription and mediates vitamin D’s genomic activities (10). The VDR gene is situated on human chromosomes 12q13-12q14, and numerous single-nucleotide polymorphisms (SNPs) have been identified, including *ApaI*, *BsmI*, *FokI*, and *TaqI* (11). The *TaqI* polymorphism arises from a mutation in the transcriber region situated in exon 9 of the VDR gene. The mutation may impact VDR posttranscriptional regulation by interacting with microRNA; it is situated in the 3′-untranslated region (UTR) of the VDR gene (12). Previous research on the relationship between VDR-*TaqI* gene polymorphism and the risk of DN in Type 2 diabetes patients has been limited and inconsistent, with some indicating a novel association and others indicating no association, emphasizing the need for additional research (13). This study sought to determine the impact of vitamin D deficiency and *TaqI* gene polymorphism on the risk of diabetic nephropathy in the Ethiopian population, providing insights into potential therapeutic targets and risk stratification approaches.

## Materials and methods

### Study participants

A total of 210 participants including 70 diabetic patients with nephropathy, 70 diabetic patients without nephropathy and 70 healthy controls participated in an age- and sex-matched hospital-based case control study. In addition to age and sex matching, we considered BMI and diabetes duration during recruitment to minimize confounding. Medication use was recorded but not matched due to variability and feasibility constraints. A hospital-based matched case-control study was carried out at Debre Tabor Comprehensive Specialised Hospital (DTCSH) from April to August 2024. At the chronic follow-up clinic (CFC), the hospital treats and monitors patients with severe chronic illnesses such as DN and T2DM. The source population included all CFC patients, while the research participants were T2DM patients without complications and DN complications who were under follow-up. The study’s controls were any non-diabetic, healthy volunteers who were available and age- and sex-matched during the research period.

### Inclusion and exclusion criteria

Type 2 diabetic patients with and without DN complications confirmed by blood glucose tests and urine albumin creatine ratio (UACR) were recruited into this study. The study included patients receiving follow-up care at CFC for at least five years. Controls were age- and sex-matched non-diabetic healthy individuals with normal blood glucose and UACR results from the same geographical location and social status. Patients with a history of hyperparathyroidism, chronic liver disease, heart failure, abnormal urinary sediment, urinary tract infection, vitamin D supplements, or chronic bacterial or viral infection were excluded. Patients unable to respond or unwilling to sign informed consent were also excluded from this study.

### Sample size determination and sampling technique

The sample size was calculated using G* power version 3.1.9.4 software by selecting an independent t-test. Since similar studies were not done in the Ethiopian population, it is calculated by considering alpha = 0.05, power (1- ß) = 0.8 (80%), effect size (d) = 0.5, and allocation ratio N2/N1 = 1. After accounting for the 10% non-response rate, the final sample size was 210 participants of both sexes, including 70 DN patients, 70 T2DM patients, and 70 healthy controls. Participants were selected by simple random sampling methods, using a table of random numbers (TRN), from all registered patients.

### Anthropometric measures

Body weight was assessed using a portable digital scale, and height was taken using a portable stadiometer. BMI was computed by dividing weight in kilograms by height in meters squared. Participants were classified as underweight (BMI < 18.5 kg/m^2^), healthy (18.5–25 kg/m^2^), overweight (25.0-29.9 kg/m^2^), or obese (≥ 30 kg/m^2^). The hip circumference was measured at the maximal circumference around the hips, and the waist circumference was measured at the umbilicus level with an inelastic measuring tape while the individuals stood upright. Obesity was defined as a waist-hip ratio (WHR) greater than 0.95 for men and more than 0.85 for women. Blood pressure was measured in the sitting position after 5 minutes of rest using digital equipment, and SBP and DBP were derived from the average of three measurements. The second and third measures were collected 5–10 minutes after the initial and second measurements, respectively. Participants were diagnosed with hypertension if their mean SBP was ≥ 140 mmHg and DBP was ≥ 90 mmHg, or if they used antihypertensive medication.

### Biochemical measures

Following an overnight fast, blood samples were collected from each participant’s median cubital vein. After centrifuging the serum, each test was subjected to an enzymatic examination of glucose, triglycerides, total cholesterol, LDL-cholesterol, HDL-cholesterol, calcium, phosphorous, and parathyroid hormone (PTH) using the Dimension EXL 200 fully automated analyzer in the DTCSH diagnostic laboratory. Participants were classified as diabetes (FBG ≥ 126 mg/dl or RBG ≥200 mg/dl) or treated with insulin or oral hypoglycemic medications; pre-diabetic (FBG 100–125 mg/dl or RBG 140–199 mg/dl); or normal (FBG <100 mg/dl or RBG <140 mg/dl). Dyslipidemia is defined as having triglycerides ≥150 mg/dl, total cholesterol ≥200 mg/dl, LDL-C ≥130 mg/dl, and HDL-C <60 mg/dl. Deficiencies of calcium, phosphorous, and PTH were diagnosed if the serum value is less than 10.5 mg/dl, 4.5 mg/dl, and 65 pg/ml, respectively. Serum vitamin D levels were evaluated using the 25-Hydroxy Vitamin D ELISA Kit, Immunodiagnostic Systems Ltd., Bolden, Tyne and Wear, UK. The sensitivity of the kit was 5.5 ng/mL (13.75 nmol/L). The intra-assay coefficient of variation (CV %) was <10%, and the inter-assay CV % was <15%. Vitamin D deficiency is defined as a 25-hydroxyvitamin D (25-OHD) level below 20 ng/mL (50 nmol/L). Random urine samples were collected from patients and normal controls to determine the urine albumin creatinine ratio (UACR). Micro albumin (mg/dl) was divided by urinary creatinine (g/dl) to determine the UACR. Diabetic nephropathy is diagnosed if UACR is ≥ 30 mg/g.

### Vitamin D receptor gene analysis

The non-enzymatic salting-out method was employed to extract DNA from EDTA-anticoagulated blood from both patients and controls. The *TaqI* genotypes were identified using forward primer 5’- CAG AGC ATG GAC AGG GAG CAA-3’ and reverse primer 5’-GCA ACT CCT CAT GGC TGA GGT CTC-3’. The genotypes for the *TaqI*’s RFLP were determined using polymerase chain reaction (PCR). The initial denaturation stage of the amplification was set to 95°C for 5 minutes, followed by 30 cycles of denaturation at 94°C for 60 seconds, annealing at 57°C for 45 seconds, extension at 72°C for 60 seconds, and final extension at 72°C for 7 minutes. The PCR-RFLP involved digesting the PCR products using 1.5 μl of *Taq1* restriction enzyme at 65°C for 3 hours. For electrophoresis, 5 μl of the digested reaction mixture was placed into a 2% agarose gel with ethidium bromide, run for 1 hour at 120 V, and observed under UV illumination. Positive and negative controls were included in each PCR run to monitor for contamination and successful amplification. To ensure genotyping accuracy, 10% of the samples were randomly selected and re-analyzed with 100% concordance with the PCR-RFLP results, confirming the accuracy of our genotyping method.

### Statistical analysis

The data was analyzed using STATA Version 17. To compare continuous variables between patients and controls, the t-test for independent samples was applied. The genotype and allele frequency distributions were compared with the chi-square test. To investigate the risk correlations of the VDD and *TaqI* polymorphisms with DN at a 95% confidence level (CL), logistic regression was used. The *TaqI* genotypes and clinical characteristics were evaluated using one-way ANOVA. A p-value of <0.05 was considered statistically significant.

## Results

### Anthropometric and biochemical characteristics

The distribution of DN patients, T2DM patients, and healthy control groups by sex and age was comparable. Of the 70 DN patients, 35 (50.00%) were male, and 35 (50.00%) were female. Of the 70 T2DM patients, 34 individuals (48.57%) were male, and 36 (51.42%) were female. Similarly, among the 70 healthy control participants, 37 individuals (52.85%) were male, while 33 individuals (47.14%) were male. The DN, T2DM, and control groups had mean ages of 59.94 ± 10.81, 59.50 ± 13.63 and 58.66 ± 8.62, respectively. Patients with DN had significantly higher average levels of SBP, DBP, and UACR but lower 25(OH)D levels compared to T2DM patients and controls (p <0.001). BMI, WHR, FBG, TC, TG, LDL, HDL, calcium, phosphorous, and PTH were not significantly different between the study groups (p > 0.05) (**Table 1**).

**Table 1:** Demographic and clinical characteristics of the study participants in Debre Tabor Comprehensive Specialized Hospital, Northwest Ethiopia, 2024.

Variables	DN (n=70)	T2DM (n=70)	Control (n=70)	P-value
Sex (M/F)	35/35	34/36	37/33	0.7368
Age (Years)	59.94 ± 10.81	59.50 ± 13.63	58.66 ± 8.62	0.9901
BMI (Kg/m^2^)	23.03 ± 4.56	22.84 ± 3.87	22.38 ± 4.18	0.9874
WHR (cm)	0.83 ± 0.13	0.83 ± 0.12	0.82 ± 0.12	0.5517
SBP (mmHg)	119.63 ± 7.04	115.15 ± 3.88	113.29 ± 3.87	<0.0001*
DBP (mmHg)	78.10 ± 4.34	77.19 ± 3.95	72.91 ± 3.90	<0.0001*
FBG (mg/dl)	108.99 ± 11.14	105.99 ± 9.13	89.00 ± 9.38	0.4872
25(OH)D (ng/ml)	16.18 ± 6.48	24.37 ± 4.24	25.14 ± 3.76	<0.0001*
Total Cholesterol (mg/dl)	174.54 ± 56.91	166.34 ± 56.71	151.44 ± 53.38	0.6929
Triglyceride (mg/dl)	133.47 ± 39.61	119.49 ± 38.62	115.56 ± 32.74	0.5001
LDL-Cholesterol (mg/dl)	94.65 ± 36.30	91.84 ± 38.48	86.64 ± 25.90	0.1388
HDL-Cholesterol (mg/dl)	61.94 ± 11.31	63.28 ± 15.39	66.54 ± 9.02	0.1820
UACR (mg/g)	76.31 ± 1.50	15.51 ± 1.65	13.28 ± 1.49	<0.0001*
Calcium (mg/dl)	8.92 ± 0.48	9.24 ± 0.45	9.41 ± 0.44	0.5116
Phosphorus (mg/dl)	3.66 ± 0.50	3.75 ± 0.42	3.78 ± 0.60	0.4164
PTH (pg/ml)	60.24 ± 15.2	61.20 ± 15.9	62.44 ± 14.3	0.1296

*Statistically significant differences at *P <*0.05.

### Association between diabetic nephropathy and vitamin D deficiency

**Figure 1:**
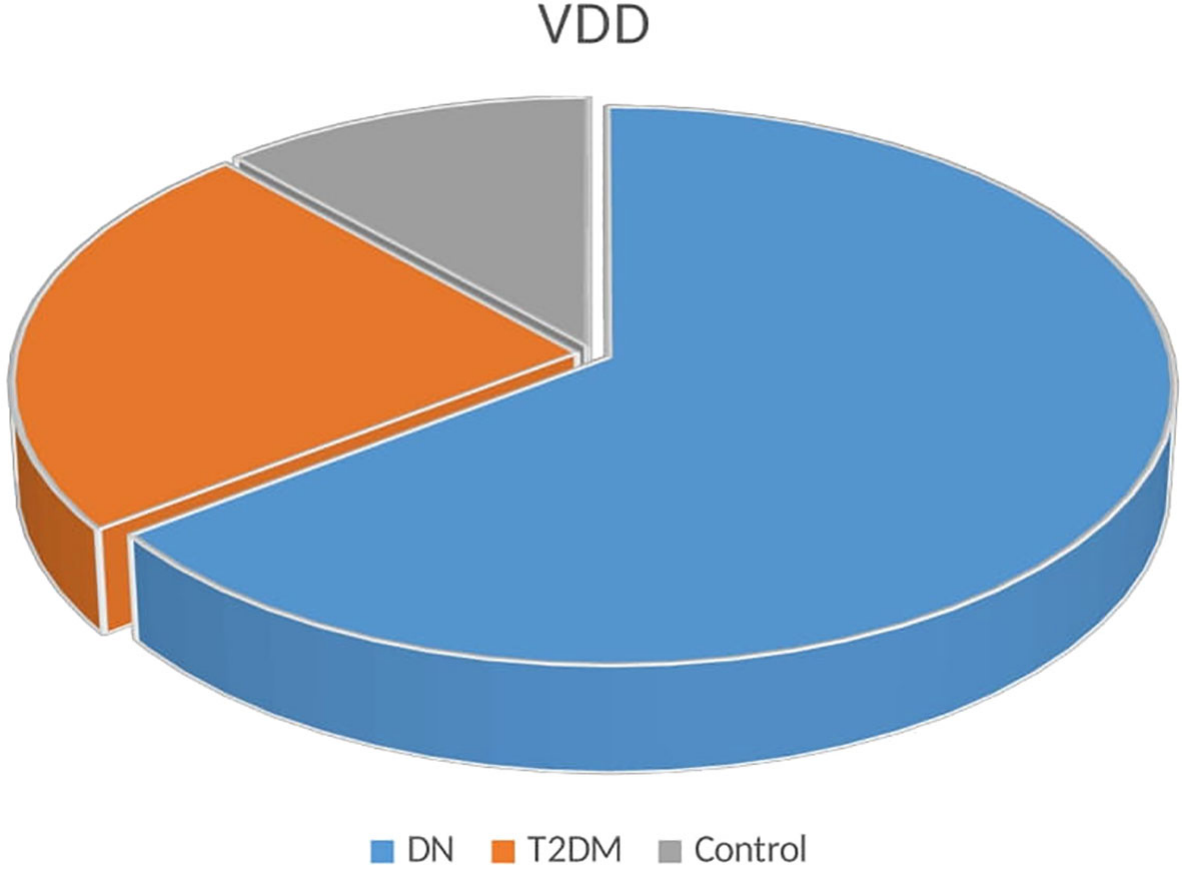
Distribution of the vitamin D deficiency among DN, T2DM, and controls.

The association between DN and VDD is illustrated in **Table 2**. A comparison of serum vitamin D levels between study participants found that patients with DN had lower levels of vitamin D than T2DM patients and healthy control groups (P <0.001). DN patients also had a larger percentage of VDD (47.14%) compared to T2DM (17.15%) controls (8.57%) (**Figure 1**).

**Table 2:** Association between diabetic nephropathy and vitamin D deficiency in Debre Tabor Comprehensive Specialized Hospital, Northwest Ethiopia, 2024.

Vitamin D Status	DN (n=70)	T2DM (n=70)	Control (n=70)	OR (95% CL)	p-value
VDD	33 (47.14%)	12 (17.15%)	6 (8.57%)	5.05 (2.03-12.53)	< 0.0001*
Non-VDD	37 (52.85%)	58 (82.84%)	64 (91.42%)	Ref	

*Statistically significant differences at *P <*0.05.

### Distribution of *TaqI* genotypes and allele frequencies

**Figure 2:**
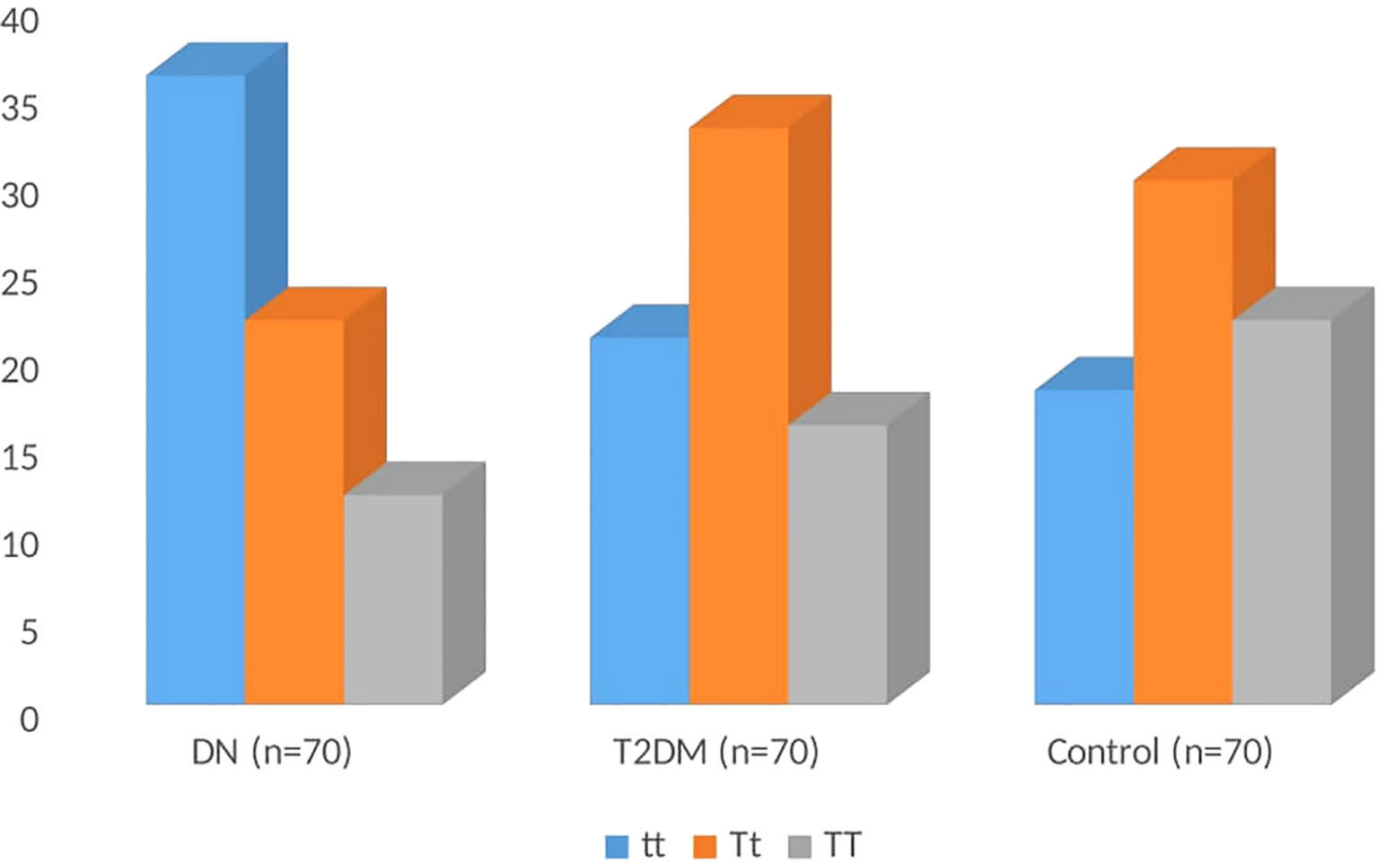
Distribution of the *TaqI* genotype among DN, T2DM, and controls.

The genotype distribution of *TaqI* gene polymorphisms is shown in **Table 3**. In DN patients, the frequencies of the tt, Tt, and TT genotypes were 51.43%, 31.43%, and 17.14%, respectively; in T2DM patients, the frequencies were 30.00%, 47.14%, and 22.86%; in controls, the frequencies were 25.71%, 42.86%, and 31.43% (**Figure 2**). The homozygous tt genotype was found to be significantly more common in DN patients than in T2DM and controls (odds ratio [OR] = 2.48; 95% CL= 1.15-5.37; P=0.020). The frequency of the t allele was substantially greater in DN patients than in T2DM and controls (OR = 1.70; 95% CL: 1.13-2.57; P=0.010). The genotype distributions were in accordance with the Hardy-Weinberg equilibrium (p > 0.05) in the control groups (X^2 = 1.37), with the t and T allele frequencies recorded at 0.47 and 0.53, respectively. This suggests that the genotype distribution in the control group supports the reliability of the genotypic data.

**Table 3:** Distribution of *TaqI* genotypes and allele frequencies of the study participants in Debre Tabor Comprehensive Specialized Hospital, Northwest Ethiopia, 2024.

Genotype	DN (n=70)	T2DM (n=70)	Control (n=70)	OR (95% CL)	P-value
tt	36 (51.42%)	21 (30.00%)	18 (25.71%)	2.48 (1.15-5.37)	0.020*
Tt	22 (31.43%)	33 (47.14%)	30 (42.86%)	1.44 (0.70-2.94)	0.316
TT	12 (17.14%)	16 (22.85%)	22 (31.42%)	Ref	
Allele Frequency					
t	94 (67.14%)	75 (53.57%)	66 (47.14%)	1.70 (1.13-2.57)	0.010*
T	46 (32.86%)	65 (46.43%)	74 (52.85%)	Ref	

*P-value <0.05 is considered statistically significant.

Ref, Reference; CL, Confidence Level; OR, Odds Ratio.

### Association between *TaqI* genotypes and clinical parameters

Fasting blood glucose, vitamin D levels, BMI, WHR, SBP, DBP, UACR, lipid profiles, calcium, phosphorous, and PTH of the study groups ‘ were analyzed to determine the *TaqI* genotypes (tt, Tt, and TT). **Table 4** summarizes the clinical parameters of DN patients, T2DM patients, and healthy controls according to the *TaqI* genotype. The genotypes in the study groups did not show a significant correlation with the above-mentioned clinical variables (P>0.05). **Table 5** shows multivariable logistic regression analyses for predictors of DN with Adjusted Odds Ratios (AOR) and 95% CL. There was no correlation of DN with sex, age, BMI, and lipid profiles (*P*>0.05). However, systolic blood pressure (OR 1.14 (95% CL 1.06–1.22), *P*=0.001) and diastolic blood pressure (OR 1.29 (95% CL 1.17–1.42), *P*<0.001) were associated with DN.

**Table 4:** Association of *TaqI* genotype with clinical characteristics in Debre Tabor Comprehensive Specialized Hospital, Northwest Ethiopia, 2024.

Variables	Genotypes			
	tt (n=75)	Tt (n=85)	TT (n=50)	p-value
BMI (Kg/m^2^)	23.08 ± 4.12	21.92 ± 4.00	23.68 ± 4.48	0.2587
WHR (cm)	0.83 ± 0.13	0.83 ± 0.12	0.81 ± 0.12	0.7300
SBP (mmHg)	117.06 ± 5.76	114.95 ± 5.37	116.29 ± 6.35	0.3514
DBP (mmHg)	77.03 ± 4.50	75.02 ± 4.69	76.40 ± 4.51	0.7464
FBG (mg/dl)	103.46 ± 18.02	100.68 ± 14.76	99.23 ± 18.07	0.7333
25(OH)D (ng/ml)	20.15 ± 7.28	22.77 ± 5.22	23.07 ± 6.38	0.1550
Total Cholesterol (mg/dl)	166.09 ± 57.66	158.75 ± 49.89	170.72 ± 64.07	0.1108
Triglyceride (mg/dl)	119.76 ± 48.76	125.37 ± 46.32	123.16 ± 37.36	0.3655
LDL-Cholesterol (mg/dl)	88.35 ± 25.69	92.70 ± 25.95	92.29 ± 27.92	0.8284
HDL-Cholesterol (mg/dl)	65.10 ± 8.94	63.44 ± 8.77	62.98 ± 9.79	0.8488
UACR (mg/g)	43.83 ± 31.31	30.39 ± 27.61	29.73 ± 26.82	0.4860
Calcium (mg/dl)	8.86 ± 0.46	8.32 ± 0.48	9.12 ± 0.45	0.6451
Phosphorus (mg/dl)	3.72 ± 0.52	3.78 ± 0.46	3.68 ± 0.58	0.5423
PTH (pg/ml)	61.84 ± 14.8	63.24 ± 16.5	65.43 ± 15.8	0.7129

*Statistically significant differences at *P <*0.05.

**Table 5:** Multivariate logistic regression analysis for predictors of diabetic retinopathy among T2DM patients in Debre Tabor Comprehensive Specialized Hospital, Northwest Ethiopia, 2024.

Variables	AOR	CL	P-value
Sex (M/F)	0.92	0.45-1.89	0.822
Age (Years)	1.00	0.97-1.04	0.829
BMI (Kg/m^2^)	1.00	0.91-1.09	0.954
SBP (mmHg)	1.14	1.06-1.22	0.001*
DBP (mmHg)	1.29	1.17-1.42	0.000*
Total Cholesterol (mg/dl)	1.01	1.00-1.01	0.087
Triglyceride (mg/dl)	1.00	0.99-1.02	0.471
LDL-Cholesterol (mg/dl)	1.00	0.99-1.02	0.724
HDL-Cholesterol (mg/dl)	0.98	0.94-1.03	0.434

*P-value <0.05 is considered statistically significant.

AOR, Adjusted Odds Ratio; CL, Confidence Level.

## Discussion

Emerging data from recent studies suggests that vitamin D may have an essential role in the development of diabetic nephropathy (DN), yet individual published investigations produced inconclusive results (14). Our study found that DN patients had significantly greater VDD compared to type 2 diabetic patients and healthy controls (**Table 2**). These results were in line with studies from Sudan (7), Egypt (14), China (15), India (16), Korea (17), Saudi Arabia (18), Iraq (19), and Iran. Although the exact mechanism by which vitamin D deficiency may be associated with an increased risk of diabetic nephropathy is not fully understood, the following factors can contribute to the development and progression of the disease. Vitamin D has potent anti-inflammatory effects, which can be diminished in deficiency, causing increased inflammation within the kidneys and damage to kidney structures (7). Moreover, vascular and endothelial dysfunction, impaired insulin sensitivity, increased oxidative stress, and podocyte injury can contribute to DN onset due to vitamin D deficiency (18). Vitamin D deficiency can impair blood pressure management and lead to microvascular consequences like DN, with the importance of each pathway varying based on individual circumstances (17). In contrast, a study undertaken in China (20) found no significant relationship between VDD and the incidence of DN, contradicting the current study’s conclusions. The contradicting finding show the practical relevance of racial diversity and could be explained by a variety of factors, including sample size disparities, study methodology, sampling bias, and matching criteria (18).

Studies on the relationship between vitamin D receptor (VDR) gene polymorphisms and diabetic nephropathy (DN) susceptibility yielded inconsistent results (21). In our study, individuals with DN showed much higher frequencies of the t allele of the *TaqI* variant than T2DM and controls, as well as significantly higher rates of tt homozygosity (**Table 3**). The findings in Egypt (22), China (21), Iran (23), and Iraq (24) populations were consistent with the current study, as the VDR *TaqI* gene polymorphism showed a significant increase in the (tt) genotype and the (t) allele in DN patients compared to the control groups. The exact underlying mechanism by which the *TaqI* polymorphism in the VDR gene might be associated with DN is still not fully understood, and the evidence for a direct link is inconclusive (24). One proposed mechanism involves reduced vitamin D action, which may be due to less efficient VDR triggered by *TaqI* gene polymorphism. As a result, vitamin D may be less efficient in promoting insulin release by pancreatic beta cells and enhancing insulin sensitivity in target tissues (21). The impact of the *TaqI* gene polymorphism may also result from its probable interaction with other genetic and environmental variables, such as those who lead unhealthy lifestyles or who are genetically predisposed to DN. In contrast, a study of populations from China (25) and India (26) found no link between the VDR *TaqI* polymorphism and the incidence of DN. The disparity between our findings and those of earlier studies could be attributed to genetic differences in the populations investigated or exposure to various environmental factors (25).

There were some limitations in this study. First, there may be bias in determining the *TaqI* genotype in DN patients due to the comparatively small sample size. Further studies with a large sample size are needed to understand the correlation between the *TaqI* gene polymorphism and the risk of diabetic nephropathy complications. Second, the absence of other VDR gene polymorphisms that directly correlate with T2DM and DN development. Future research should expand the genetic analysis of diabetic nephropathy by including additional vitamin D receptor polymorphisms, such as *BsmI*, *ApaI*, and *FokI*, alongside the *TaqI* polymorphism. This broader genetic approach will provide a more comprehensive understanding of how various genetic variations influence DN risk and vitamin D metabolism. Third, our findings may be specific to the studied Ethiopian population and may not be directly generalizable to other ethnicities or non-hospitalized populations. Future investigations in diverse populations are necessary to validate our findings.

## Conclusions

In conclusion, our study found that vitamin D deficiency was substantially more common in DN patients than in T2DM patients and healthy controls, verifying the association between low vitamin D levels and increased diabetic nephropathy risk in the Ethiopian population under study. Furthermore, there was a possible link between diabetic nephropathy and the TaqI gene polymorphism tt genotype and t allele, suggesting that the *TaqI* gene polymorphism might be used as a potential genetic risk marker for diabetic nephropathy detection. To completely understand the link between the VDD, *TaqI* gene, and DN, more research with a larger sample size is needed.

## Data Availability

The original contributions presented in the study are included in the article/supplementary material. Further inquiries can be directed to the corresponding author.
